# Development and evaluation of an emergency department serious game for undergraduate medical students

**DOI:** 10.1186/s12909-024-06056-z

**Published:** 2024-09-27

**Authors:** Alexandra Aster, Christopher Hütt, Caroline Morton, Maxwell Flitton, Matthias Carl Laupichler, Tobias Raupach

**Affiliations:** 1 Institute of Medical Education, University Hospital Bonn, Venusberg-Campus 1, 53127 Bonn, Germany; 2 Department of Anesthesiology and Intensive Care Medicine, University Hospital Bonn, Bonn, Germany; 3 Yellow Bird Consulting Ltd, London, UK

**Keywords:** Serious game, Gamification, Medical education, Clinical reasoning, Game development, Usability, User experience

## Abstract

**Background:**

Serious games are risk-free environments training various medical competencies, such as clinical reasoning, without endangering patients’ safety. Furthermore, serious games provide a context for training situations with unpredictable outcomes. Training these competencies is particularly important for healthcare professionals in emergency medicine.

**Methods:**

Based on these considerations, we designed, implemented, and evaluated a serious game in form of an emergency department, containing the features of a virtual patient generator, a chatbot for medical history taking with self-formulated questions, artificially generated faces based on an artificial intelligence algorithm, and feedback for students. The development process was based on an already existing framework resulting in an iterative procedure between development and evaluation. The serious game was evaluated using the System Usability Scale and the User Experience Questionnaire.

**Results:**

The System Usability Scale provided a substantial result for the usability. In terms of the user experience, four scales yielded positive results, whereas two scales yielded neutral results.

**Conclusion:**

The evaluation of both usability and user experience yielded overall positive results, while simultaneously identifying potential areas for improvement. Further studies will address the implementation of additional game design elements, and testing student learning outcome.

## Background

Serious games are known to be safe, cost and time effective learning environments, which are used in various application areas, including the healthcare sector [1–3]. Besides, there is an urgent need to create digital learning environments [4] to motivate learners, as more and more users are digital natives and it is assumed that serious games are particularly helpful for enhancing the learning outcomes of this user group [5]. In the healthcare sector, serious games provide a risk-free environment for tasks that might put patients at risk or have an unpredictable outcome, while maintaining a high fidelity [6, 7]. Particularly noteworthy is the psychological fidelity, which reflects the extent to which the experience of psychological factors (e.g., stress) are simulated similarly to the real environment [8]. Hence, serious games seem to have a significant effect on learning outcomes with regard to patient safety, and help students feel more confident and eased in real-life situations afterwards [9].

For contexts such as medicine, healthcare professions, or even patient education the use of serious games proves to be an effective teaching method in terms of learning outcomes [6, 10]. According to the frequently referenced definition by Michael and Chen [11, p. 21] serious games are defined as “games that do not have entertainment, enjoyment, or fun as their primary purpose”. Therefore, the fundamental difference is that serious games primarily focus on learning objectives and simultaneously contain entertaining elements, whereas the sole goal of entertaining games is to elicit amusement in the players [12] without having a primary learning objective. Concerning digital serious games, Laamarti, Eid [12, p. 4] provide the refined definition of “serious games as an application with three components: experience, entertainment, and multimedia […]”. Conclusively, serious games have to be contrasted with the related concepts ‘gamification’ and ‘game-based learning’. Contrary to the fully fledged serious game, ‘gamification’ only implicates the addition of game elements to non-game contexts [13]. ‘Game-based learning’ instead can be understood as the pedagogical approach of incorporating games into curricula with serious games being its operationalization [14].

In the particular context of medical education, serious games offer the opportunity to gather knowledge as well as abilities in a “safe space” without the risk of endangering the health of real patients [6, 15]. Moreover, serious games allow for fostering and strengthening non-technical skills (e.g. communication or coping with stress) or knowledge about patient safety in medical students [9, 16]. Strengthening these and other non-technical skills (e.g. teamwork) is of great importance for successful work in dynamic environments, such as those found in an emergency department [17]. Serious games are already used to strengthen and enhance teamwork between disciplines such as medical and nursing undergraduates in the emergency room [18]. Besides, the development of distinct and crucial clinical reasoning competencies (i.e. proposing a suspected diagnosis as well as initiating necessary investigations and appropriate therapies, [19]) is essential for physicians in emergency departments. Clinical reasoning competencies comprise a holistic view of the patient, including the surrounding factors, as well as the adaptation to altering circumstances [20]. Since medical and healthcare professionals are confronted with difficulties and biases during clinical decision-making, it is necessary to further teach and train those competencies [20]. It has already been shown, that serious games can work as an effective method for training clinical reasoning in medical students [21] as well as in other healthcare professions education such as nurse education [22].

It is essential to find an appropriate learning environment for teaching highly relevant skills to medical students in order to prepare them for working in an emergency department. The implementation of a valid emergency department simulation in face-to-face teaching (e.g. with simulated patients) is hardly viable, hence the idea to create a suitable serious game arose. Fostering learning achievements while simultaneously evoking fun and entertainment depends fundamentally on a structured development and evaluation process of the serious game [23]. Therefore, Olszewski and Wolbrink [24] proposed a structured framework for serious game development in medical education, which we applied for the development of our presented serious game. The framework consists of three iterative phases, namely Preparation & Design, Development, and Formative Evaluation. The current development stage of our serious game is in the transition between phase two and three, more precisely in iteration loops between usability testing and the ongoing development. Usability is therefore understood as defined in ISO 9241-11 as “the extent to which a system, product or service can be used by specified users to achieve specified goals with effectiveness, efficiency and satisfaction in a specified context of use“ [25, p. 269]. User Experience is also understood according to the definition by ISO 9241 − 210 as “a person’s perceptions and responses that result from the use and/or anticipated use of a product, system or service.” [26, p. 1]. The following section describes the endeavor and the current development status in further detail. Finally, an evaluation process with medical students and its results are presented.

## Development and construction of the serious game

In this paper, we summarize the development and evaluation process of the serious game “DIVINA” and provide a prospect on further steps and possible studies. The term “DIVINA” is an acronym of the German ‘DIgitale VIrtuelle NotAufnahme’, which translates to ‘digital virtual emergency department’.

### Design and development

The development stage started in 2020 with a design phase. A design team was put together with a software developer who is also a physician (from the commercial company leading the development) and two additional physicians. The concept here was to use co-design where stakeholders (in this case medical educators and doctors) were involved from the beginning in the design of the product. Up to three medical students assisted the design team and helped enter disease-specific data (see below). Since medical students are supposed to be the prospective users, there is a growing claim to include them in a participatory way in the development process [27]. It is recommended to involve end-users in many steps in the development process to take into account user expectations and facilitate a proper user experience [28]. In addition, students can support game development with regard to adherence to design principles (e.g. goals, feedback, rewards, as well as more general narratives and aesthetics) from a user perspective [29]. Thus, the combined perceptions of creators and end-users yield a holistic approach.

Later during the ongoing process, a psychologist joined the design team to supervise the design process and provide support regarding psychological background knowledge. The focus was on design elements to promote learning processes and outcomes. Our interdisciplinary approach to game development (i.e. software developer, physicians, students, psychologists) was in line with current recommendations as it holds a number of advantages [16]. One of the main advantages of our team is the software developer simultaneously being a physician. Hence it is unlikely that information or expert knowledge gets lost due to communication difficulties or misconceptions [30].

### Educational content and learning objectives

Clinical reasoning, according to Kassirer [19], covers on the one hand the competencies of formulating a suspected diagnosis based on the patients’ medical history and contrasting it against differential diagnoses. On the other hand, it is about initiating necessary investigations to confirm the suspected diagnosis and initiating an appropriate initial therapy. Fostering the competencies of clinical reasoning represents one of the main learning objectives of the serious game presented here. Another important non-technical skill is coping with stress. Nevertheless, the right decisions regarding the urgency of patients’ symptoms and consequently the order of treatments should be made. Thus, a further learning objective is prioritizing patients and initiating the necessary medical procedures under time pressure.

The player is placed in the situation of being a physician currently working at an emergency department. The game starts with a variety of patients appearing on a dashboard (representing the waiting room or arrivals via ambulance). Subsequently, students have to admit patients according to the perceived urgency of the situation. Once a patient has been assigned to a treatment room, students can perform the following actions: taking a medical history via a chatbot, measuring vital signs, arranging diagnostic tests including interpretation of findings, performing a physical examination, ordering laboratory tests, prescribing medication and further measures. Before patients can be discharged or transferred (e.g. to their home, a normal ward or to an intensive / intermediate care unit), students have to complete discharge notes and choose a diagnosis. After discharging or transferring a patient, students are provided with static feedback on the specific disease of that patient. Constantly incoming new patients in the dashboard with different presenting complaints and symptoms create time pressure.

Diseases are not restricted to one particular area of medicine (e.g., cardiology, gastroenterology or gynecology). The structure of the database facilitates the addition of diseases related to all specialties lending themselves to be included in a virtual accident and emergency department. This leads to a current number of 50 implemented diseases.

### Target group

Undergraduate medical students are the primary target group of this digital teaching resource. However, the game is not solely intended for asynchronous self-directed learning. Instead, gaming ‘sessions’ containing specific diseases need to be created by teachers and made available to students during synchronous learning sessions. Depending on curricular requirements and opportunities, actual gaming sessions may be accompanied by teachers via online communication services (lends itself for larger groups) or in a small-group setting.

### Structure of the game

**Fig. 1:**
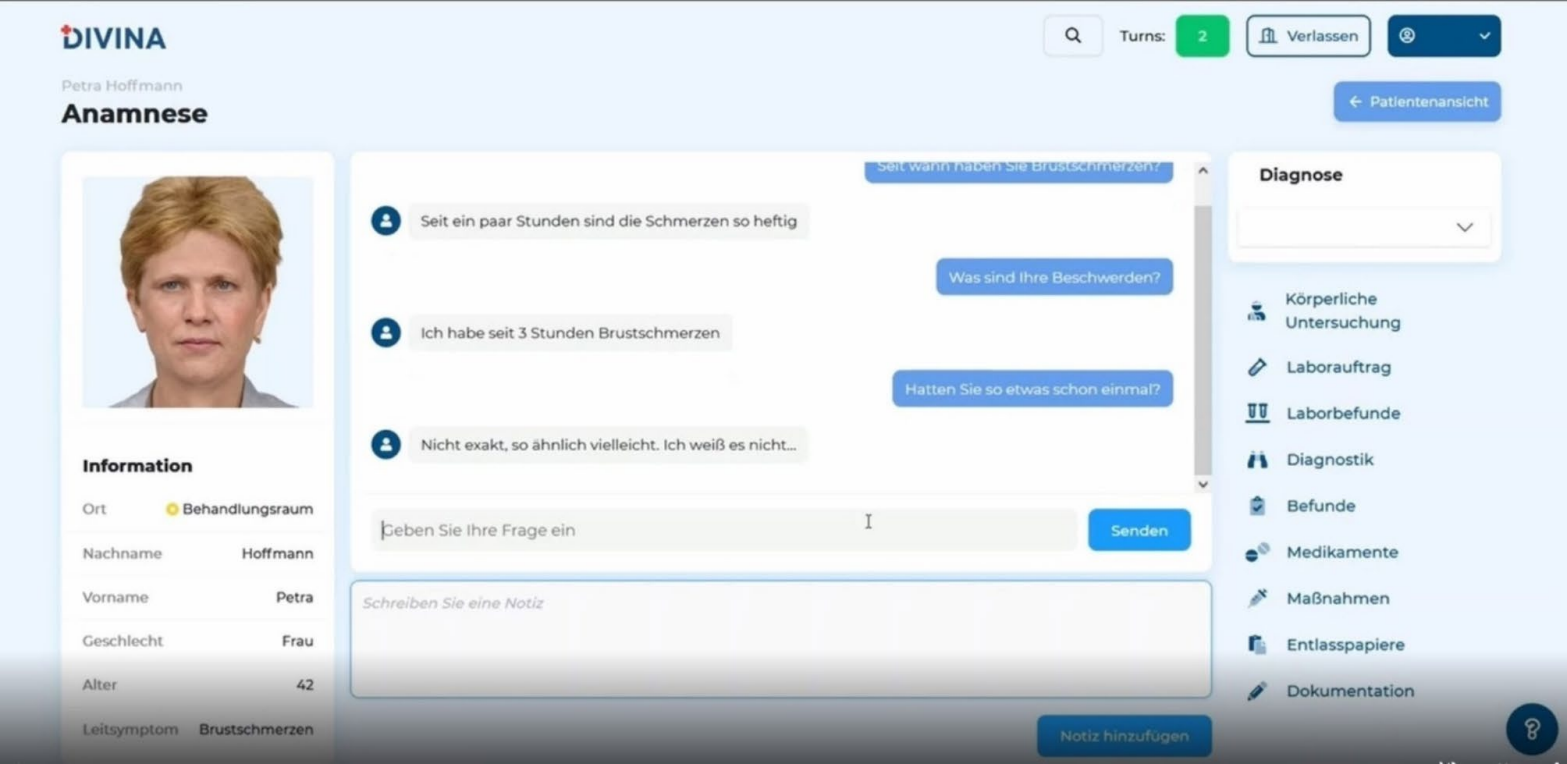
DIVINA interface in German. On the left side, information about the patient, including given name, name, age, and leading symptom as well as the AI generated face is depicted. The central element is the chatbot. On the right side, all other necessary actions can be found. The information contained in the picture is meant for demonstration purposes only. Neither the image, nor the depicted information represent real patient data

The serious game was programmed in Python, Rust and React, with a data pipeline set up via GitHub. The serious game is playable in every common browser. In the interest of maximal flexibility for users, and in order to avoid disruptions due to server overload, the design team decided to use a 2D- rather than a 3D-Design. Currently, the serious game is available to all German medical students via a DFN (Deutsches Forschungsnetz) login that recognizes students at all German medical schools. For a presentation of the game interface, see Fig. 1.

The first innovative feature of this digital resource is the way in which virtual patients (VP) are generated. To build a VP suffering from a specific disease an algorithm refers to the deposited epidemiological data. Due to the disease generation stemming from epidemiological probabilities, each VP is unique that prevents easy identification of the disease. Thus, it is secured that the learning process is not only driven by recognition, as students cannot easily recall a VP from an earlier gaming session. At the same time, the VPs provide recurring learning scenarios and offer the possibility for regular knowledge repetition as it is recommended for learning environments in general, but especially for simulated ones [31]. In addition, artificial faces generated by a style-based generator using an artificial intelligence (AI) algorithm [32] complement the VPs and illustrate the disease by matching the symptoms. Faces appear either as healthy, or pale, reddish, cyanotic, or jaundiced. This enhances the psychological fidelity of the environment as it allows replicating the real emergency department environment to a certain extent [8]. It is also noteworthy, that the usage of AI-generated faces prevents data privacy problems accompanying the usage of real patient faces.

Another innovative feature is the chatbot that offers students the opportunity to enter questions related to the patients’ anamnesis on which the chatbot provides automatically generated answers. Whereas most other solutions offer a question menu to choose from, the chatbot in DIVINA allows for freely formulated questions. This feature sets DIVINA apart, as students have the opportunity to learn history taking by considering questions and formulating them on their own. The questions asked by students can relate to the presenting complaint, associated symptoms, history of presenting complaint, past-medical history, as well as drug and social history. Furthermore, the chatbot offers the chance for teachers to analyze how students approach the history taking, yielding potential for further teaching on this topic. Due to the necessity of asking self-generated questions and considering all relevant topics, the chatbot aims to foster clinical reasoning competencies.

## Evaluation

### Methods

In order to test the usability and user experience of the proposed serious game, we collected data during a study in summer term 2022 with third-year medical students (*N* = 146) participating. The local Institutional Review Board reviewed the study protocol in winter term 2021/2022 (application number 34/8/21). The study was embedded in a six-week cardiology and pneumology module at Goettingen Medical School. Students participating in the module played the game in two sessions lasting 90 min each. The content of the respective game sessions were taught in the formal teaching sessions the week before. The evaluation of usability and user experience formed the conclusion of the study, for which students gave their informed consent beforehand.

Usability was assessed using the 5-point Likert-scaled System Usability Scale (SUS, [33]) and user experience was evaluated via the User Experience Questionnaire (UEQ, [34]). The SUS offers a result scale between 0 and 100 with an average score set around 68 [35]. Regarding the UEQ, two opposing extreme response options were rated on a seven-stage scale ranging from − 3 to + 3, with − 3 being the negative extreme, 0 neutral, and + 3 the positive extreme [36]. Taken together the UEQ comprises six different scales, namely Attractiveness (i.e. overall impression), Perspicuity (i.e. easy familiarization), Efficiency (i.e. efficient task solving), Dependability (i.e. feeling of control regarding interaction), Stimulation (i.e. exciting and motivating usage), and Novelty (i.e. innovation of the product) [36]. All participants answered the System Usability Scale, whereas, for the sake of brevity, only one-half of the cohort answered the UEQ for DIVINA. The second half were asked to comment on a different learning resource that was not the focus of this paper. In addition, free text questions were provided in which participants could indicate what they already liked or disliked about the game and where they see further room for improvement.

## Results

According to Brooke [33] the inverted items were recoded manually. As items could be omitted, only a total number of 127 complete datasets were assessed for the SUS and revealed a mean of *M* = 59.19. Hence, the response rate for the SUS was 85%. The result of the SUS falls within the first quartile spanning from 30.0 to 62.6 and is below the average score of 68 [35].

The UEQ comes along with an excel data analysis tool, which was used for the evaluation of the data. Since missing values could be included in the appraisal, 76 datasets were used for the evaluation of the UEQ, leading to a response rate of 100%. Broken down according to the scales, the results were as follows: Attractiveness (*M* = 0.94, *SD* = 1.29, *95% CI* [0.65, 1.23]), Perspicuity (*M* = 1.12, *SD* = 1.02, *95% CI* [0.89, 1.34]), Efficiency (*M* = 0.05, *SD* = 0.98,

*95% CI* [-0.18, 0.27]), Dependability (*M* = 0.73, *SD* = 0.86, *95% CI* [0.54, 0.93]), Stimulation (*M* = 0.82, *SD* = 1.32, *95% CI* [0.52, 1.11]), Novelty (*M* = 1.25, *SD* = 0.88, *95% CI* [1.06, 1.45]). The excel data analysis tool also provided benchmarks that concerned different commercial products. Compared to these benchmarks the results regarding DIVINA were bad for the scales efficiency and dependability, below average for the scales attractiveness, perspicuity and stimulation, and good for the scale novelty.

Since students are supposed to be the end-users of the serious game, we invited them to provide us with additional free text feedback in order to conclude further room for improvement. Most of all, students suggested to further improve the server capacity as well as improvements of the chatbot. Besides suggestions for improvement, students also emphasized the opportunity to apply knowledge in a safe learning environment without the risk of endangering patients’ lives. Furthermore, students appreciated receiving feedback that we already implemented based on a pilot iteration with a different cohort of medical students.

## General discussion

In this overview of the development and evaluation process, we co-operated with a software company to introduce a serious game representing a schematic simulation of an emergency department. Besides the introduction of the serious game, a first usability and user experience testing was conducted. To support the achievement of the learning outcomes, the serious game is already equipped with some supporting features, namely feedback, AI-generated faces, and a free text chatbot.

The evaluation of the current stage revealed promising preliminary results while simultaneously highlighting areas for further improvement. The usability score can be interpreted as substantial and falls in the first quartile of the SUS ratings, which can be set between 30.0 and 62.6 according to Bangor [35]. In other words, the result at hand represents a marginally low acceptability. Since usability defines how systems can be used effectively, efficiently and satisfactorily to achieve predefined goals, it can also be understood as the operability of a system. The low usability score might be due to the user interface or inadequate server capacity, as students remarked some issues. The user interface might have been confusing for students in terms of the arrangement of elements, as they sometimes reported problems finding the needed elements. Another problem area that might have led to the low usability rating became apparent in the users’ feedback texts. Students perceived the chatbot as an inadequate tool for taking a medical history, as it sometimes answered incoherently. Although the chatbot answered incoherently and students did not always felt like getting satisfactory answers, it is nevertheless an innovative feature that enhances the psychological fidelity of the serious game. It can be argued that the chatbot enhances the psychological fidelity, as the students have to ask self-formulated questions, just like in a real-life emergency department. In addition to the chatbot, the AI-generated faces enhance the psychological fidelity and the game’s educational value, as it can be assumed that students remember the patients better by their faces compared to their leading symptom. However, future studies have to investigate these assumptions.

In terms of the user experience, the scales attractiveness, perspicuity, stimulation, and novelty yielded positive ratings, whereas the scales efficiency, and dependability received neutral ratings. The positive ratings of attractiveness and novelty showed that participants liked the use of the serious game, and perceived the design as innovative. In addition, perspicuity and stimulation were positively evaluated, as the use of the serious game was easy to learn, but also exciting and motivating. Efficiency received a neutral rating, possibly due to the additional effort while solving the tasks. Another scale, which received a neutral score, was dependability, as participants might not have felt the game to be predictable and therefore did not feel like having control over the interaction with the game. While both scales were rated with a neutral rather than a negative score, they indicate potential opportunities for improvement. These usability and user experience ratings were to be expected based on the user interface and playability of the game, but should be still used to improve the game in further iterations.

We finished the first stage of the development framework proposed by Olszewski and Wolbrink [24] since we already assembled a suitable interdisciplinary team, transferred the medical concepts, produced the essential content, and mapped the learner experience. From now on, the serious game will pass a continuous loop between development and formative evaluation. Based on evidence, all relevant evaluative findings will be implemented in further development iterations, which will in turn be evaluated again. The next step in the development process is the implementation of effective game design elements based on psychological learning theories and accompanying the development with further studies. Additionally to the implementation of further game design elements, already existing elements like the chatbot as well as the general user interface will consistently be improved. At each stage, student feedback is heeded, and helpful suggestions will be incorporated into the game evidence-based. Since this is a dynamic project, we will assist the software company to further implement new disease data to meet the demands of undergraduate medical students and their teachers. It is also conceivable to realize the opportunity of asynchronous self-directed learning with this serious game. Furthermore, it is also conceivable to extend the game in the long term to postgraduate medical students, residents, or other healthcare professions working in an emergency department.

### Limitations

To ensure a concise evaluation following the game session, only half of the students completed the UEQ for DIVINA. Consequently, the interpretability of UEQ data in comparison to SUS data may be constrained due to the unequal population size. It is important to note that the UEQ, designed primarily for the commercial market, compares results of the individual scales to benchmark values oriented towards such products. Additionally, the study occurred at a single time of measurement, introducing potential limitations related to situational events like server capacity issues, as already reported by students. Replicating the evaluation at several times may circumvent these issues. The study, being part of a mandatory event, implies that participation was largely driven by extrinsic factors, potentially influencing the evaluation results. Repeating the evaluation in a voluntary study setting, where participation is likely driven by intrinsic motivation, could offer different insights. However, whether participation is influenced by intrinsic or extrinsic motivational factors should be a focus of future studies.

## Conclusion

Serious games provide a risk-free learning environment that is highly valuable in the context of medical education. Therefore, a serious game presenting a virtual emergency department was developed and evaluated in terms of its usability and user experience. Overall, the evaluation yielded positive results and identified potential areas for further improvement. The results of the evaluation will be integrated into the consistent development of the serious game to offer medical students a valuable learning source for education in the field of emergency care.

## Data Availability

The datasets used and/or analyzed during the current study are available from the corresponding author upon reasonable request.
